# Medical Society of the State of Pennsylvania

**Published:** 1856-07

**Authors:** Henry Carpenter, Alfred L. Kennedy

**Affiliations:** Recording Secretary; Recording Secretary


(To be continued.)

---

### MEDICAL SOCIETY OF THE STATE OF PENNSYLVANIA.

The Medical Society of the State of Pennsylvania met, pursuant to adjournment, in the City of Philadelphia, at the Hall of the University (Department of Arts,) on Wednesday, May 28, 1856, and was called to order at 11 o'clock by the President, Dr. J. S. Carpenter, of Schuylkill.

Dr. J. B. Biddle, in behalf of the local Committee of Arrangement, suggested the following hours of meeting, which, on motion, were agreed to:—

Wednesday, from 11 to 1 o'clock, and from 3 to 5; and on Thursday and Friday, from 9 to 1. On the afternoon of Wednesday, the members were invited to visit the Academy of Natural Sciences, and on Thursday afternoon the Girard College and the Institution for the Blind, and on Friday afternoon the Society would be conveyed by steamboat to the Lazaretto, where the country members would be entertained as the guests of the Philadelphia delegation.

On motion of Dr. H. Carpenter, Drs. W. Worthington, B. S. Janney, and C. W. Parrish, were appointed a committee to examine and report on the credentials of members.

On motion of Dr. R. P. Thomas, *Resolved*, That the Society now hear the Address of the President.

The President, Dr. J. S. Carpenter, then delivered the Annual Address.

On motion of Dr. John Schrack, the thanks of the Society were tendered to the President for his excellent Address, and a copy was requested for publication in the *Transactions*.

The Committee on Credentials reported the following gentlemen to be duly accredited by their respective Societies.

The names of those who were in attendance are marked by an asterisk.

*Beaver county*—Drs. *Oliver Cunningham, Beaver; *David E. Marquis, Freedom.

*Berks county*—Drs. *Wm. Greis, Reading; *Ed. Wallace, Reading; J. M. Matthews, Coxtown; C. H. Hunter; *Elias Kitchen, Douglassville; Wm. Moore, Womelsdorf; J. H. Wanner, Kutztown.

*Bucks county*—Drs. O. P. James, Doylestown; S. N. Evans, Johnsville; Philip H. Grier, Ottsville; Saml. Carey, Quakertown.

*Bradford county*—Drs. A. K. Axtell, Troy; *Geo. F. Horton, South Asylum.

*Cambria county*—Dr. *John Loman, Johnstown.

*Carbon county*—Dr. *D. K. Shoemaker, Rockport.

*Chester county*—Drs. *Wilmer Worthington, West Chester; Isaac Thomas, West Chester; *Septimus A. Ogier, Frazer; *Caleb Swayne, London Grove; *W. W. Townsend, Chatham; W. D. Hartman, West Chester; *John P. Edge, Downingtown; Jacob Price, West Chester; *Isaac R. Walker, West Chester; *C. W. Parish, Marshalton.

*Delaware county*—*Drs. Manly Emanuel, Marcus Hook; *Gurdon B. Hotchkin, Media; *Chas. J. Morton, Chester; *R. H. Smith, Media.

*Lancaster county*—Drs. John Reum, Hempfield; Abm. Eshleman, Strasburg; O. Cassidy, Lancaster; *Benj. Rohrer, Columbia; J. K. Rant, New Providence; *John L. Atlee, Lancaster; *Jos. Gibbons, Intercourse; Abm. Shellar, Mount Joy; J. L. Atlee, Jr., Lancaster; Thos. Ellmaker, Lancaster; Augustus Withers, Safe Harbor; Jos. H. Lefevre, Paradise; *Henry Carpenter, Lancaster; *J. A. Ehler, Lancaster.

*Lawrence county*—Drs. J. S. Cosset, New Castle; A. P. Dutcher, Enon; *D. Leasure, New Castle.

*Lebanon County*—Drs. *Geo. P. Lineaweaver, Lebanon; *B. F. Schneck, Lebanon; *P. B. Mish, Cornwall.

*Montgomery County*—Drs. *Wm. Corson, Norristown; E. K. Beaver, Worcester; Benj. Johnson, Norristown; J. A. Martin, Whitemarsh; *John Schrack, Shannonville; Cyrenius Williams, Norristown; C. Shoemaker, Jenkintown; *Wm. R. Ramsey, Norristown; *Hiram Corson, Plymouth Meeting.

*Northampton County*—Drs. *J. R. Ludlow, Easton; Breckenridge Clemens, Easton.

*Philadelphia county*—Drs. James Ash, Germantown; *Franklin Bache, Philadelphia; *T. Hewson Bache, do.; *John Bell, do.; John B. Biddle, do.; *Thos. F. Betton, Germantown; *Joseph Cason, Philadelphia; *Benj. H. Coates, do.; *D. Francis Condie, do.; *Gouv. Emerson, do.; *Robt. A. Given, do.; *John D. Griscom, do.; *W. W. Gerhard, do.; *Lewis P. Gebhard, do.; *Paul B. Goddard, do.; *Henry Hartshorne, do.; *Edward Hartshorne, do.; *Nathaniel L. Hatfield, do.; *Samuel L. Hollingsworth, do.; *Samuel Jackson, (Prof.) do.; *Saml. Jackson, (Spruce St.,) do.; *Benj. S. Janney, do.; *Wilson Jewell, do.; *Edgar Janvier, Richmond; W. L. Knight, Philadelphia; *Saml. Lewis, do.; *E. F. Leake, Frankford; *R. La Roche, Philadelphia; Chas. D. Meigs, do.; *Wm. Mayburry, do.; *J. H. B. McClellan, do.; *John Neill, do.; *Geo. W. Norris, do.; *John M. Pugh, West Philadelphia; *Lewis Rodman, Philadelphia; *Isaac Remington, do.; *Henry Y. Smith, do.; *Anthony C. Stocker, do.; *J. Henry Smaltz, do.; *Geo. B. Wood, do.; *Caspar Wister, do.; *Thos. H. Yardley, do.; *Jacob S. Zoons, Kensington.

*Schuylkill county*—Drs. *B. F. Shannon, Schuylkill Haven; *A. H. Halberstadt, Pottsville; *J. F. Treichler, McKenasburg; *A. Heger, Pottsville; *J. H. Wythes, Port Carbon.

*Susquehanna county*—Dr. L. A. Smith, New Milford.

*Washington county*—Drs. *Robt. Davidson, West Alexander; *J. W. Wishart, Washington.

Which report of the Committee on Credentials was, on motion, accepted.

On motion of Dr. Worthington, Dr. H. K. Neff, a member of the Huntingdon County Medical Society, was received as a Delegate from that Society, and Dr. Luke Granger, of Lawrenceville, Tioga, a county in which no Medical Society at present exists, was invited to participate in the proceedings of the session.

On motion of Dr. H. Carpenter, the reading of the minutes of the last session was dispensed with.

Dr. W. Jewell offered the following resolution, which was, on motion, agreed to.

*Resolved,* That a committee be appointed, consisting of one from each county in attendance, to nominate officers of the Society for the ensuing year, and that each county delegation choose its own representatives on said Committee.

On motion of Dr. A. E. Stocker, the Committee above constituted were empowered to nominate the additional members of the Committee of Publication, and to designate the place of meeting of the Society in 1857.

The following gentlemen were announced by their respective delegations as constituting the Committee to nominate officers for the ensuing year:—

Drs. Oliver Cunningham, Beaver Co.; William Greis, Berks Co.; Philip H. Greir, Bucks Co.; George F. Horton, Bradford Co.; D. K. Shoemaker, Carbon Co.; S. A. Ogier, Chester Co.; G. B. Hotchkin, Delaware Co.; H. K. Neff, Huntingdon Co.; J. A. Ehler, Lancaster Co.; D. Leasure, Lawrence Co.; G. P. Lineaweaver, Lebanon Co.; John Schrack, Montgomery Co.; J. R. Ludlow, Northampton Co.; Caspar Wister, Philadelphia Co.; B. F. Shannon, Schuylkill Co.; J. W. Wishart, Washington Co.

A letter from Dr. P. Cassidy, one of the Vice Presidents, was read, regretting his inability, from sickness, to be present and participate in the proceedings of the session.

A communication was received from Dr. R. E. Rogers, Dean of the University, inviting members to visit the Wistar and Horner Museums. Invitations were also received from the authorities of the Girard College and the Academy of Natural Sciences.

On motion, the invitations were accepted, and acknowledgments tendered to the Institutions severally.

The report of the Susquehanna County Medical Society was presented, read, and, on motion, referred to the Committee of Publication.

The report of the Blair County Medical Society was presented, and the reading proceeded with until the hour of adjournment.

Adjourned to 3, P. M.

*Afternoon Session, May* 28, 1856.

Society met agreeably to adjournment. President in the chair, roll called, and minutes of the morning session read and approved.

The reading of the report of the Blair County Medical Society commenced this morning, was concluded, and the report referred to the Committee of Publication.

The report of the Lawrence County Medical Society was presented, read, and on motion, referred to the Committee of Publication.

Dr. Condie reminded the Society of a resolution now in force, by which reports of more than eight pages were required to be accompanied with an an abstract of their contents, not exceeding that length.

The report of the Bradford County Medical Society was presented.

On motion of Dr. Condie, that so much of the report be read as has been prepared by the Chairman of the Sanitary Committee of that county, which was agreed to, and the portion read accordingly.

Dr. Jewell objected that this course was not in accordance with the resolution above cited by Dr. Condie, and moved that the report be referred back to the delegation from Bradford County, with instructions to prepare an abstract for presentation on to-morrow, which motion was agreed to.

The Treasurer, Dr. R. P. Thomas, presented and read his report, which was, on motion, referred to an Auditing Committee, consisting of Drs. Maybury, H. Hartshorne and Worthington.

Dr. Condie offered the following resolution:

*Resolved,* That a committee be appointed to examine the minutes of the last session, and report such items of unfinished business as may appear therein, in order that the same may be acted upon at the present session; which resolution was adopted, and the committee, consisting of Drs. Condie, Atlee and Greis, appointed.

The report of the Lebanon County Medical Society was presented, and on motion, read and referred to the Committee of Publication.

Adjourned to 9 o'clock A. M. to-morrow.

—

*Morning Session, May* 29, 1856.

The Society met pursuant to adjournment. President in the chair. On calling the roll, thirty-one members answered to their names.

Minutes of preceding meeting were read and approved.

A communication was received from the Mutual Life Insurance Company, New York, on the subject of Vital Statistics.

Dr. Betton moved that the communication be referred to a committee of three, with instructions to report during the present session of the Society—which motion was agreed to, and Drs. Betton, Condie and Jewell were appointed the committee.

Dr. Worthington presented the following preamble and resolutions, which had been adopted by the Chester County Medical Society at the stated meeting held 29th ult.:

*Whereas,* The medical profession, in the discharge of its various and responsible duties to individuals and the public, is more severely taxed for charitable and benevolent purposes than any other profession, business or occupation; and *whereas*, the constant aim of its members has been to enlarge the sphere of its usefulness, by the organization of societies and the publication of their *Transactions*, for the dissemination of important information; and *whereas*, much good may result not only to the profession, but to the people generally, by a more liberal circulation of these *Transactions;* Therefore,

*Resolved*, That in the opinion of this Society the *Transactions* of the Medical Society of the State of Pennsylvania ought to be published by the Legislature of the State, thereby relieving the profession of this pecuniary burthen, and giving a more extended publicity to the labors of a useful institution.

*Resolved*, That our delegates be instructed to bring this subject before the State Society at its next Annual Meeting, with a view of making application to the next session of the Legislature for such legislation as may be deemed necessary to attain the object contemplated.

Dr. Condie moved that the communication be referred to a committee of three, with instructions to draw up a memorial embodying the sense of the resolutions, and to lay the same before the Legislature of the State at its next session; which motion was agreed to, and Drs. Condie, Betton and Worthington were appointed the committee.

A communication was received from the officers of the Hospital for Diseases of the Chest, inviting the members of the Society to visit that institution during their stay. On motion of Dr. Condie, the invitation was accepted.

Dr. Kennedy, in behalf of the Committee on Forms for County Reports, presented the following report, which was accepted, and the resolution appended was adopted:

PHILADELPHIA, May 27, 1856.

*To the President and Members of the Medical Society of the State of Pennsylvania:*

The Committee on Forms for County Reports beg leave to state that agreeably to the direction of the Society at last meeting, they have had printed and circulated copies of the Form as presented by them and approved by the Society.

The importance of full, accurate and uniform sanitary statistics, cannot be over-estimated, and professional as well as public duty, demands that such statistics be obtained and published.

The Committe anticipate that the experience of the coming year will suggest modifications on the Forms for the Reports by which the end contemplated may be more fully secured, and therefore request the passage of the following resolution:

*Resolved*, That the Committee on Forms for County Reports be, and they are hereby instructed, to continue the circulation of the printed Forms as heretofore, and to report to this Society, at the next session, any modification of the Form which the experience of the year may show to be desirable.

Signed, ALFRED L. KENNEDY,
D. FRANCIS CONDIE,
S. L. HOLLINGSWORTH.

The Committee to Examine Minutes of last session, reported the following items of unfinished business:

Report of Committee on Vaccination, etc., p. 11. (See Vol. IV. p. 10.)

Resolutions of Chester County Medical Society, p. 12. (See Vol. IV. p. 17.)

Report of Committee on Quack Medicines, etc., p. 15.
" " Adulteration of Drugs, p. 15.
" " Extension of Organization, p. 16.
" " Death of Dr. J. P. Heister, p. 16.
" " Death of Drs. Richards and A. S. Baèr, p. 16.

Respectfully submitted,

Signed, D. FRANCIS CONDIE,
JNO. L. ATLEE.
WM. GRIES.

On motion, the report was accepted, and the committee discharged.

An abstract of the report of the Bradford County Medical Society was presented and read, and, on motion, the report was referred to the Committee of Publication.

The report of the Huntingdon County Medical Society was presented and read, and, on motion, referred to the Committee of Publication.

The names of the committee to nominate officers for the ensuing year being called for by Dr. Ehler, were read.

On motion of Dr. Wister, Dr. Worthington was appointed a member of the nominating Committee, for Chester County, in the place of Dr. Ogier, now absent.

The Nominating Committee then retired for the purpose of preparing their report.

Dr. Biddle presented and read the Report of the Board of Censors.

On motion of Dr. J. L. Atlee, the minutes of the Board of Censors were also read. Dr. Atlee enforced his motion by a reference to the importance of sustaining the Code of Ethics, to the just and manly course pursued throughout the case by the Philadelphia Society and the Board of Censors, and to the value of the decision as a precedent for future action. The motion being agreed to, the minutes of the Board were read, and the report was accepted and ordered for publication, as follows:

The Board of Censors of the 1st and 2d Districts of the "Medical Society of the State of Pennsylvania," have the honor to submit to the Society the following report of its action in the matter of the appeal of John Coleman Morgan, M. D., from the Philadelphia County Medical Society.

The Board of Censors convened pursuant to notice given, in the city of Reading, on the 24th July, 1855; present, Drs. J. B. Biddle, of

Philadelphia, Hiram Corson, of Montgomery, and Edward Wallace of Berks; and a quorum being present, the Board organized by the appointment of Dr. Biddle as Chairman, and Dr. Wallace as Secretary.

The appellant, Dr. Morgan, duly appeared in person before the Board, and the Philadelphia County Medical Society was represented by Drs. D. F. Condie, B. H. Coates, A. E. Stocker and H. Hartshorne.

The Record of the Philadelphia County Medical Society, herewith subjoined, having been read, it appeared that Dr. John Coleman Morgan, having been charged by Dr. G. Emerson, before the Censors of the Philadelphia County Medical Society, with being publicly associated with irregular practitioners in the faculty of an institution known as the Penn Medical University, and the Censors, after due investigation of the charge, having found it to be substantiated, the said Dr. Morgan was on the 18th April, 1855, at a stated meeting of the Philadelphia County Medical Society, expelled therefrom.

Dr. Morgan having appealed from this action of the Philadelphia County Medical Society, was duly heard in his defence before the Board of Censors of the 1st and 2d Districts of the Medical Society of the State of Pennsylvania, convened at the time and place above mentioned, and the Committee of the Philadelphia County Medical Society was also heard in reply.

Upon the conclusion of the addresses of the appellant and appellees, the Board of Censors unanimously adopted the following resolution:

*Resolved*, "That the action of the Philadelphia County Medical Society be confirmed."

And in accordance with the provisions of the Constitution, in reference to the duties of its Censors, the Board of the 1st and 2d Districts respectfully submit this report of their action, in the case of this appeal.

Signed, J. B. Biddle,
Hiram Corson,
Edward Wallace.

29th May, 1856.

Dr. F. G. Smith presented and read the Report of the Committee of Publication, to wit:

The Committee of Publication respectfully report, that 750 copies of Vol. V. of the *Transactions* were printed and distributed in proportion to the representation of the various County Medical Societies, at an aggregate expense of $250 64, full details of which will be found in the Treasurer's report.

There are now in possession of the Society the following numbers of each volume:

Vol. I., 2 copies; Vol. II., 32 copies; Vol. III., 30 copies; Vol. IV., 26 copies; Vol. V., 20 copies.

But two complete sets are now in the possession of the Society. These, the Committee recommend, should be retained and bound for the use of the Society.

A complete index to all the volumes published having been appended to the last No. of the *Transactions*, the Committee think that this is the proper time to suggest an alteration in the mode of publication, based upon the fact that the amount of matter contributed annually is not enough to form a volume of sufficient size to be bound separately. They beg, therefore, respectfully to propose the following resolution :

*Resolved,* That the Committee of Publication be instructed to issue the *Transactions* hereafter *in parts,* to be paged continuously ; and that when as many are issued as will form a volume of about 500 pages, that an index and title be appended to the last part.

As no provision has been made by the Constitution for the organization of the Committee of Publication, your present Committee further recommend the adoption of the following resolution :

*Resolved,* That hereafter, the Committee of Nomination be instructed to propose one of the Committee of Publication, who shall act as Chairman, and who shall be a resident of the place at which the *Transactions* are to be published.

All of which is respectfully submitted.

FRANCIS G. SMITH, *Chairman.*

On motion, the report was accepted and the resolutions thereunto appended were adopted.

The Committee to Audit the Treasurer's account report having examined the vouchers and found the account correct.

On motion, it was resolved, that the reports of the Treasurer and of the Auditing Committee be accepted, and the Committee discharged.

*Robert P. Thomas, Treasurer, in Account with the Medical Society of the State of Pennsylvania.*

DR.

1855.
June 1st. To balance received from Dr. West, former Treasurer, . 32 00

1856.
May 28. For contributions received from the Medical Societies of the following Conties, viz. :

For 1854.

| | | |
|---|---|---|
| Alleghany | 19 00 | |
| Bradford | 7 00 | |
| Cambria | 12 00 | |
| Lebanon | 12 00 | |
| Perry | 8 00 | |
| Susquehanna | 8 00 | |
| | | 66 00 |

And for 1855.

| | | | | |
|---|---|---|---|---|
| Alleghany | 15 00 | Huntingdon | 15 00 | |
| Berks | 20 00 | Lancaster | 37 50 | |
| Blair | 8 00 | Lebanon | 8 00 | |
| Bradford | 8 00 | Montgomery | 17 50 | |
| Bucks | 10 00 | Northampton | 12 50 | |
| Cambria | 12 00 | Schuylkill | 17 50 | |
| Chester | 25 00 | Philadelphia | 81 00 | |
| Delaware | 10 00 | Washington | 10 00 | |
| | | | | 300 00 |
| | | | | $398 00 |

May 28. To Cash balance . . . . . . . . . . $30 43

Cr.

| | | |
|---|---|---|
| 1855. | | |
| June 1. | By cash paid T. K. & P. G. Collins, balance for printing the *Transactions* of 1854 . . . . . . . . | 87 32 |
| 1856. | | |
| Jan. 16. | By cash paid T. K. & P. G. Collins, for printing the *Transactions* of 1855 . . . . . . . . . . | 250 64 |
| " | " cash paid Messrs. Collins for printing circulars for the Corresponding Secretary . . . . . . | 6 00 |
| May 26. | " cash paid T. H. Yardley, Corresponding Secretary, for envelopes and postages paid by him . . . . | 6 50 |
| " | " Treasurer's bill for postages . . . . . . . | 2 25 |
| " 27. | " A. L. Kennedy, Secretary, for advertising meetings, postages, and printing blank forms . . . . . | 14 86 |
| " 28. | " Balance forward . . . . . . . . . . . | 30 43 |
| | | $398 00 |

E. E. Philadelphia, May 28th, 1856.

ROBERT P. THOMAS, *Treasurer.*

The Medical Societies of Erie, Lycoming, Mercer and York Counties, reported by my predecessor last year as being delinquent for 1853 and previous years, still remain in arrears. In consequence of this, no copies of the *Transactions* have been forwarded to those Counties.

I have the pleasure to report that all the contributions for 1854 have been paid up.

For 1855, those of Franklin, Lehigh, Mifflin, Perry and Susquehanna County Societies, amounting in all to $37 50, remain unpaid, but these will probably be liquidated in a short time.

The Society is now entirely out of debt, and from the foregoing statement, it will be seen there are $30·43 in the Treasury subject to its order.

Robert P. Thomas, *Treasurer.*

An abstract of the report of the Chester County Medical Society was presented and read, and, on motion, the report was referred to the Committee of Publication.

An abstract of the report of the Montgomery County Medical Society was presented and read, and, on motion, the report was referred to the Committee of Publication.

An abstract of the report of the Philadelphia County Medical Society was presented and read, and, on motion, the report was referred to the Committee on Publication.

On motion of Dr. J. L. Atlee, Resolved, that a committe of five be appointed from the Philadelphia County Delegation, to memorialize the city authorities on the subject of Vaccination; which motion, after a brief discussion, in which Drs. Atlee, Bell and Condie participated, was agreed to, and the following gentlemen were constituted the Committee: Drs. Jno. Bell, B. H. Coates, H. Hartshorne, Wm. Mayburry and J. Remington.

On motion of Dr. Bell, Dr. Condie was added to the Committee. The Committee appointed this morning, on the communication from the Mutual Life Insurance Company, presented the following report, which was, on motion, accepted, and the Committee discharged:—

Your Committee, having carefully considered the several propositions submitted to this Society for information in respect to the vital statistics of the State of Pennsylvania, and certain points bearing upon the value of life within our bounds, beg leave to report that they place a high estimate upon the importance of the subjects embraced in the propositions alluded to, and their faithful solution. As, however, it is scarcely to be expected that any committee appointed by this Society would be willing or able to devote the labor and time necessary to prepare satisfactory answers to the four propositions contained in the communication of the New York Insurance Company; your Committee would therefore suggest that, provided said insurance company appoint, in this State, a Commission to collect the various materials essential to a solution of the propositions on which information is requested, it would not be improper, and would, perhaps, be advisable, that this Society should appoint a Committee to assist, as far as possible, the said Commission in the performance of their duties.

T. F. Betton,
D. F. Condie.

The report of the Medical Society of the City of Reading and County of Berks was presented and read, and, on motion, referred to the Committee of Publication.

On motion of Dr. J. L. Atlee, Dr. Isaac McKinney, a member of the Lycoming County Medical Society, and now present, was received as a Delegate from that Society.

Dr. T. H. Yardley, Corresponding Secretary, presented the following ing report, which was read, and, on motion, accepted:—

"The Corresponding Secretary of the Pennsylvania State Medical Society reports that, agreeably to a resolution adopted at the last meeting of the Society, he "addressed a circular to the prominent physicians in those portions of the State in which no County Medical Society exists, urging upon them the importance of organizing such society without delay.

Of the sixty-three counties in the State, only twenty-four have ever given evidence of the existence of a County Medical Society, leaving thirty-nine in which it is presumed no such organization exists. These circulars were sent in numbers varying from two to thirty to each county.

Much difficulty was found in ascertaining the name and address of physicians in many parts of the country, and many very estimable gentlemen may have escaped attention; I am under great obligations to Professor Gilbert, of the Pennsylvania Medical College, and Professor Rand, of the Philadelphia Medical College, both of whom furnished a list of names.

Dr. Hays, Editor of the *Medical News*, and Dr. Hollingsworth, Editor of the *Medical Examiner*, also greatly aided in giving publicity to the circular, by inserting it in their respective journals.

Communications have been received from thirteen counties, viz.:

Beaver, Butler, Clarion, Dauphin, Elk, Fayette, Greene, Lawrence, Tioga, Union, Venango, Wayne and Westmoreland.

All the writers express a desire to organize a Society in their county, and to all of them a copy of the *Transactions* of the last year was sent, to aid them in their efforts.

It is obvious, from the remarks of these correspondents, that the great barrier to the organization of County Societies, in many parts of the State, is Sec. 2d, Article 6, of the Constitution of the State Society, which says—'no one shall be admitted as a member of a County Society, unless he is either a graduate in medicine of some respectable medical school, or has a license to practice from some board recognized by the State Society, or has been a practitioner for at least fifteen years.' Several allude to this; and one gentleman writes—'this rule will exclude some of the most talented and best educated physicians in our county. Some of them have been my students; and though I am a graduate, and have been more than fifteen years in practice, yet I would not be willing to join a Society which must exclude those deserving young men.' 'These gentlemen, after graduating in literary colleges, attended one course of medical lectures, and then commenced practice, intending to attend another course, and graduate at their earliest convenience; but they soon became so situated as to render it impossible to leave their practice and home long enough to accomplish that object.'

Th. H. Yardley,
*Cor. Sec. State Med. Society.*

N. B. I have received the Constitution of the Medical Society of Beaver County, approved by the Censors of that district.

T. H. Y."

Dr. H. Carpenter in behalf of the Committee on Vaccination and Vaccine Virus, stated that circumstances had prevented the Chairman of the Committee from preparing their report, as well as from being present at this session of the Society.

Dr. Remington moved that the Committee be discharged, and a new committee appointed, which motion prevailed; and Drs. H. Carpenter, G. Emerson, and C. W. Parish, were constituted the committee.

Dr. Condie, in behalf of the Committee on Quack Medicines, presented and read the following report, which was accepted, the Committee discharged, and the resolution appended to the report was adopted.

"*To the Medical Society of the State of Pennsylvania:*

To the undersigned, a Committee appointed at the last annual session of this Society, was referred the entire subject involved in a resolution offered by Dr. Carpenter, of Schuylkill County, in the following words, namely: 'That the members of this Society, and the profession generally, be strongly advised to use their influence with apothecaries and druggists to induce them to discontinue the purchase and sale of patent and quack medicines, and that the profession be recommended to furnish their patronage to those only who comply with this request;' and, also, an amendment proposed to said resolution by Dr. John Neill, of Philadelphia, by adding after the words 'quack medicines,' the words, 'and patented instruments.'

Whether, in referring to your committee the whole subject connected with this resolution, and the proposed amendment, it was intended that they should simply report their views as to the policy of the adoption by the Society of the first, with or without the latter, or whether it was expected that they should enter into an examination of the entire question as to the propriety of the use of and traffic in patent and quack medicines, and patented instruments, and the duty of this society, and of the medical profession generally, to use their influence to put a stop to the traffic by apothecaries and druggists, and the propriety of physicians withholding their patronage from such as shall persist in it, does not very clearly appear from the terms of the resolution under which your committee was appointed.

The question, in all its bearings, is one, unquestionably, of considerable interest. That the use of and traffic in patented and quack medicines and nostrums, is at once improper and dishonorable, is, we believe, the universally received opinion of the medical profession. It is so pronounced by the Code of Ethics, adopted by the American Medical Association, and accepted by every State Medical Society throughout our Union; while the Society of our own State, to exhibit in the strongest light its disapproval of the use of and traffic in these medicines, has, by its constitution, excluded from affiliation with it every county society that admits to membership any one who sells or deals in them, or in any way encourages their employment.

With this open and formal denunciation, on the part of the medical profession, by the mouth of its recognized representatives, the duty of its members to discountenance the traffic in patent and quack medicines and nostrums on the part of apothecaries and druggists, and to exert every honorable means within their power to induce them to relinquish it, must be apparent. And so soon as apothecary stores, conducted by competent and skilful pharmaceutists, shall be established in the different sections of our larger cities and inland towns and villages, from which all patented remedies, quack remedies, and nostrums of every description are excluded, it will become the duty, as well as the policy of physicians, to confine their patronage exclusively to them. But whether, before the establishment of stores of this character, it would be wise or prudent to refuse sending a prescription to an apothecary merely from the fact that objectionable articles are kept for sale by him will, perhaps, be questioned. A physician engaged in extensive practice, and, at the same time, desirous of devoting as many hours to study as will enable him to keep up steadily with the onward progress of his profession, cannot, with justice to himself and to his patients, dispense with the services of the pharmaceutist for the compounding of his prescriptions. The separation of the duties of the apothecary from those of the medical practitioner is attended with advantages which only imperative necessity should be allowed to sacrifice. By a general and unwavering concert of action among the members of the medical profession, there would be no difficulty of excluding every objectionable article from the shelves of the druggist and apothecary, but until such a concert of action can be obtained, your committee believe it would be impolitic to advise the physician to dispense with the services of an otherwise skilful and reliable pharmaceutist because he keeps patented medicines for sale, when the services of no other is to be obtained against whom the same objection does not apply.

While there is an almost unanimous agreement among the members of our profession as to the impropriety of physicians prescribing, or apothecaries dealing in patented medicines or nostrums, there still are some who boldly advocate not only the propriety of physicians employing patented instruments, but of being themselves the proprietors of them, and who, of course, approve of the traffic in them by the apothecary.

It is no more than just, they argue, that he who invents a useful instrument or apparatus, should have secured to him the reward of his ingenuity and skill. But, if it be just in reference to an instrument or apparatus, why is it not equally so in reference to the discovery of any new remedial agent, or of some admirable combination of the medicines in common use? Does the invention of the first evince more genius, a higher degree of professional knowledge, or closer and more profound habits of observation, than the discovery of the last? The fact is, that not a single argument has been adduced to prove the propriety of the interposition of a patented right to prevent the free enjoyment of the one, that does not equally prove the same thing in reference to the other.

Far be it from us to withhold the reward which is justly due, whether from him who increases the value and efficiency of the materia medica, or from the one who makes a valuable addition to the armamentarium chirurgicum; we should certainly, however, not agree that the just reward due to either is a right to derive a pecuniary profit by taxing such of his professional brethren as would desire to avail themselves of the discovery or invention for the relief of those suffering from the effects of an accident, or from disease, with the power to forbid its use by such as are unwilling to pay the tax.

Your committee would desire that a higher and more enduring reward should be conferred, one more in accordance with the high-minded, honorable, and unselfish principles which should ever characterize the legitimate practitioner of the healing art, and a reward that no one who has succeeded in augmenting the remedial and curative resources of his profession can fail to receive.

We deny the right of a physician or surgeon, by any means, legal or other, to prevent or curtail the free enjoyment, by his professional brethren, of whatever discovery or improvement he may have the good fortune to make in the means adapted to relieve or remedy the accidents or diseases to which the human frame is liable. The claims of humanity, and the duties he owes to his profession, alike forbid him to monopolize or conceal the results of his observation, knowledge, or skill. He is bound, by his responsibilities as a man, and as a practitioner of a liberal art, to freely communicate these results, that, in the hands of others, they may become, perchance, the means of a still higher and more important advance in medical or chirurgical knowledge and practice.

They who claim the right to secure to themselves, by the interposition of a patent right, a pecuniary recompense from the profession for their inventions and discoveries, forget that they were themselves indebted to the profession, in the first instance, for the very knowledge by the application of which they have been enabled to improve or increase the therapeutic or surgical means for carrying out the great objects of the healing art. They lose sight of the fact that they have been allowed to avail themselves freely of the rich fund of medical and surgical knowledge that has been accumulated by the joint labors of the most distinguished practitioners of all ages, and of all countries; and that this privilege is enjoyed by them only on the implied condition that, on their part, they will as freely contribute the results of their skill and industry, to still further extend and enrich the common fund by which they have been so largely benefitted.

But, even could it be shown to be right and proper for medical men to employ and deal in patented instruments, still the question remains as to the propriety of encouraging, or countenancing the indiscriminate sale of these instruments by apothecaries.

If the latter were restricted to the dispensation of their wares only on the order or prescription of a physician, the keeping in their stores patented instruments would, so far as the apothecaries alone are concerned, be a matter of very little moment. But, as they feel themselves at liberty to keep for sale anything and everything upon which they can make a profit, and to vend their heterogeneous wares to whomsoever they can induce to become purchasers, and to employ all the usual means to augment the number of their customers, there is a danger that, when patented instruments form a part of the stock in trade of the apothecary, they, as is the case with the entire list of patent medicines and nostrums, will be procured and applied by those who are totally incompetent to decide upon the cases to which the respective instruments are adapted, or the manner in which they are to be adjusted, in order that the object they are intended to effect shall be best secured. And there is the more danger of this, from the fact that these instruments are always widely advertised, and their infallibility boldly proclaimed through the public papers; and, still further to encourage their popular use, each instrument is usually accompanied by printed directions for its application by unprofessional purchasers. Besides all this, the apothecary commonly feels himself warranted, however small may be his modicum of surgical knowledge and skill, to recommend these instruments in cases to which he deems them applicable, or even himself to superintend their application.

We are acquainted with instances, and similar ones must have fallen under the notice of other physicians, in which individuals affected with hernia, with uterine displacement, or with spinal deformity, or who have supposed themselves to be so affected, have, upon the faith of a newspaper advertisement, or the recommendation of an apothecary, purchased a patented instrument, supposed to be adapted to remedy their particu lar ailment, from the use of which they have experienced an increase of suffering, and even serious permanent injury. These occurrences cannot be avoided so long as patented instruments are kept for sale, to whoever may desire to purchase them, in the shop of the apothecary.

Your Committee are, therefore, convinced that under no circumstances whatever can it be proper to countenance the sale of these instruments by apothecaries and druggists, and they would recommend the adoption, by the Society, of the following resolution:—

*Resolved*, That the members of the Medical Society of the State of Pennsylvania, and the profession generally, be recommended to use their influence with the druggists and apothecaries of their respective neighborhoods, to induce them to discontinue the purchase and sale of patent and quack medicines, and patented instruments; and that physicians be recommended to withhold, as far as practicable, their patronage from such apothecaries and druggists as persist in the sale of the articles indicated.

D. Francis Condie.
Jas. S. Carpenter.
Sept. A. Ogier.

*Philadelphia, May* 26, 1856.

Dr. Jos. Carson, in behalf of the Committee on Resolutions of the Berks Co. Society on the Adulteration of Drugs, asked leave to be continued, which request was, on motion, granted.

Dr. Wister, in behalf of the Committee to nominate officers of the Society for the ensuing year, reported the following nominations:—

*President*,—R. La Roche, Philadelphia County.

*Vice-Presidents*,—Wilmer Worthington, Chester County; Samuel Jackson, Philadelphia; Oliver Cunningham, Beaver; George F. Horton, Bradford.

*Recording Secretaries*,—Alfred L. Kennedy, Philadelphia County; Septimus A. Ogier, Chester.

*Corresponding Secretary*,—Thomas H. Yardley, Philadelphia County.

*Treasurer*,—Robert P. Thomas, Philadelphia County.

*Censors—First and Second Districts*,—J. B. Biddle, Philadelphia County; John L. Atlee, Sr., Lancaster; W. Isaac Thomas, Chester; Hiram Corson, Montgomery; Chas. Marten, Lebanon; R. E. James, Northampton; G. F. Horton, Bradford; Edward Wallace, Berks.

*Censors—Third and Fourth Districts.*—Jos. A. Landis, Blair County; J. B. Luden, Huntingdon; Luke Granger, Tioga; Thomas Wood, Lycoming; Jos. Henderson, Mifflin; J. H. Case, Perry.

*Censors—Fifth and Sixth Districts.*—J. W. Wishart, Washington County; Oliver Cunningham, Beaver; D. Leasure, Lawrence; Jno. T. Ray, Mercer; C. F. Perkins, Erie.

*Delegates to the American Medical Association.*—J. S. Carpenter, Schuylkill County; Robert B. Davidson, Washington; Thos. F. Betton, Philadelphia; H. K. Neff, Huntingdon; J. A. Ehler, Lancaster; Jno. Shrack, Montgomery; Edward Wallace, Berks; D. K. Shoemaker, Carbon; C. F. Schneck, Lebanon; Manly Emanuel, Delaware; Philip H. Grier, Bucks; David E. Marquis, Beaver.

Additional members of the Committee of Publication, Francis G. Smith, Saml. Lewis, T. Hewson Bache.

The Committee also reported that they had selected West Chester as the place of meeting in 1857.

On motion, the report was laid on the table until to-morrow.

Dr. Condie presented and read the following report and resolutions on the death of Dr. J. P. Heister, which were, on motion, adopted and the Committee discharged.

Whereas the Pennsylvania State Medical Society feels it to be a duty which it owes to itself, to the profession, and to the families of such of its members as may be removed from it by death, and at the same time, a mournful privilege, the exercise of which it would not willingly relinquish, to place on permanent record the expression of its estimate of the worth of the departed, and of the loss it has sustained in their demise; therefore be it

*Resolved*, That in inscribing upon the list of its deceased members the name of Dr. J. P. Heister, it was with a deep sense of the severe deprivation it had experienced in common with the medical profession of Berks County and the entire State. Exemplary in all his relations as a citizen, and a Christian; well instructed as a physician, and, as such, faithful in the discharge of his professional and ethical duties, and ever ready to promote the extension of medical knowledge and the elevation of the character and standing of the profession throughout our State and country; as a member of this Society, faithful and zealous in the discharge of his obligations, and as its presiding officer dignified and impartial in the discharge of its functions; the deceased, while living, commanded the esteem of his fellow members, by whom his memory will be ever cherished with profound respect.

*Resolved*, that this Society have deeply commiserated with the family and friends of the deceased in their bereavement, and most heartily join with his professional brethren of the County of Berks in the testimonial of regard for his memory.

D. Francis Condie,
J. H. Seltzer.

Dr. A. Heger offered the following resolutions:—

*Resolved*, That a standing Committee of five, of which the present Committee on Forms shall be three, be appointed, whose duty it shall be to take charge of the department of Meteorology; give the necessary directions to observers; organise a proper system of observations throughout the county societies, and report yearly to the Association.

*Resolved*, That each county society be and is hereby requested to appoint a similar committee, consisting of one or more observers, as the topographical formation of each county may call for; to make such observations as the above Committee may direct, or they may deem proper; and to report yearly on or before the 1st Monday in March to the chairman of the Committee of this Society.

On motion of Dr. Kennedy the resolutions were adopted, and Drs. Heger and Wythes appointed the additional members of the Committee on Forms under the resolution.

In the absence of Dr. Gemmil, Chairman of Committee on the Extension of County Organizations, Dr. J. B. Biddle reported that the Committee had discharged the duty assigned to them by preparing and circulating an appeal in behalf of such organization among the members of the profession in those counties of the State in which no societies existed. On motion that the Committee be discharged, it was so ordered.

Dr. G. Emerson submitted the following resolutions :—

*Resolved,* That a committee be appointed to procure a supply of vaccine matter fresh from the original source, or recently obtained, for the purpose of distribution among the members of the several county societies of the State.

*Resolved,* That the said Committee be authorised to charge for the virus so distributed such an amount as will cover any expense incurred in procuring and distributing the virus.

On these resolutions a most able and interesting discussion ensued and was continued until the hour of adjournment, by Drs. Emerson and Condie and Wishart, Prof. Jackson and Drs. Gries and Bell.

Adjourned until to-morrow at 9 o'clock.

*Friday Morning, May* 30, 1856.

The Society met, pursuant to adjournment, at 9 o'clock. Dr. J. S. Carpenter, President, in the chair. Present thirty-seven members.

The minutes of yesterday's session were read and approved.

A communication was received from Dr. A. E. Stocker, Recording Secretary of the Philadelphia County Medical Society, enclosing a list of the officers and members, and certifying that Drs. J. C. Morgan, and S. S. Brooks, had been disfellowshipped by that Society.

Dr. Worthington called up the subject of Vital Statistics, reported upon yesterday, and urged the propriety of the Society's acting at once on the suggestion contained in the report, viz: That a committee be appointed to confer with the Mutual Life Insurance Company, N. Y., or its agent.

A discussion ensued on the general subject of Vital Statistics, in which Drs. Worthington, Condie, Jewell, Kennedy, Wythes, and Stocker participated; by all of whom the importance of the thorough and systematic collection of reliable sanitary data was fully recognized; the speakers differing, however, as to the means to be pursued in obtaining those data, and especially as to the propriety of appointing a committee to confer with the company aforesaid. The question was disposed of by the adoption of the following resolution, proposed by Dr. Worthington :—

*Resolved,* That a committee be appointed to confer with Life Insurance Companies, or other public bodies, or with any committee which they may appoint, on the subject of Vital Statistics.

Drs. Worthington, Condie, and H. Carpenter, compose the committee under the resolution.

The following report and resolution, on the descease of Drs. C. O.

Richards and E. S. Baer, of Lancaster County, were submitted by Dr. H. Carpenter, and on motion adopted:—

The members of the Medical Society of the State of Pennsylvania, having adopted the very appropriate and laudable rule of placing permanently upon its records such testimonials as may be submitted, commemorative of the worth of its deceased members, the undersigned committee, appointed to present resolutions expressive of the feelings of the Society upon the loss they have sustained in the decease of Drs. C. O. Richards and E. S. Baer, of Lancaster County, submit the following:—

The deceased were both active, intelligent, and successful physicians; enjoying in a large degree the confidence and esteem of the community in which they lived; they were removed from the sphere of their labors while yet in the vigor of youth and heighth of usefulness; and, from their ability and skill in their profession, their exemplary character as citizens, and their many amiable and endearing qualities as companions and friends, their decease will leave a void that will long be felt by their numerous friends and acquaintances; therefore—

*Resolved*, That this Society have heard, with unfeigned regret, of the death of their late fellow members—Drs. Richards and Baer; and while they deeply deplore this dispensation of an allwise Providence, they will ever hold in lively remembrance their many virtues, and sincerely tender to their families and friends their heartfelt condolence and sympathy, in their melancholy bereavement.

Signed, HENRY CARPENTER,
G. M. GEMMILL.

Dr. H. Hartshorne presented the following preamble and resolutions:—

*Whereas*, the annual reports to this body, of the County Medical Societies, notwithstanding their ability and value, contain, on account of the paucity of the members contributing to them, only a partial account of the sanitary experience of the year; and whereas system and convenience in the method of reporting may facilitate the obtaining of more complete returns, therefore—

*Resolved*, That a committee of three be appointed by the President, to report upon the practicability of issuing a *form of record*, sanctioned by the Society, for the purpose of obtaining uniform returns from the members of the different County Medical Societies, in regard to the *amount of disease* occurring under their care: and that the same committee report also upon the propriety of passing the following resolutions:—

*Resolved*, That this Society recommend to the different County Medical Societies of the State, the adoption of a uniform method of recording and reporting the amount of disease, as well as of mortality, occurring within their limits each year.

*Resolved*, That it be recommended to the several County Medical Societies, to make it one condition of the eligibility of any member as delegate to the State Medical Society, that he shall have furnished an abstract, or summary, of the principal diseases occurring under his care, to the appropriate committee of the county in which he resides, for the year preceding, or a reason for not having so done.

*Resolved,* That no member of this Society shall, hereafter, be eligible as delegate from it to the American Medical Association, who has not furnished some account of the principal diseases passing under his treatment during the year, to the appropriate committee of his County Medical Society, unless a sufficient reason be afforded for the omission in each case.

*Resolved,* That in the sanitary reports of the different County Medical Societies, the committees be requested to mention, hereafter, the names of all those members who have contributed to their materials.

On motion of Dr. Jewell, the preamble and resolutions were adopted, and Drs. H. Hartshorne, Jewell, and Wythes were appointed the Committee.

Dr. Stocker moved to amend Art. VI, Section 6, of the Constitution, so as to read as follows (the amending clause being in italics):—

Any member of a County Society who is censured or expelled shall have a right to appeal to the Censors of the district, *provided the said appeal be filed within three months after the date of said act of censure or expulsion.*

Which amendment was unanimously agreed to.

The following preamble and resolutions were offered by Dr. R. P. Thomas, and, on motion of Dr. J. L. Atlee, the consideration of them was postponed until the next annual session of the Society:—

*Whereas,* the frequent meetings of the American Medical Association are attended with much inconvenience and expense to the members, and, from the limited time afforded to the chairmen of committees having special subjects under consideration, their reports must necessarily be limited or imperfect; therefore,

*Resolved,* That in the opinion of this Society, the advancement of the best interests of the profession, and the better development of the history and treatment of diseases, as modified by local peculiarities, will be enhanced by the adoption of a longer interval in the meetings of the American Medical Association.

*Resolved,* That the delegates from this Society to the American Medical Association, be requested to bring the subject before the next annual meeting of that body, and recommend the substitution of triennial for annual sessions.

Dr. G. B. Hotchkin offered a series of resolutions calling for the publication of a condensed history of the Society, from its origin to the present time, with the Constitution, Code of Ethics, and the various resolutions now in force; the said history to be revised once in five years. The resolutions were, on motion, laid on the table.

Dr. A. K. Gaston presented the report of the Committee on the Resolutions of the Chester County Society (see printed *Transactions,* vol. IV., p. 17). On motion that the report be printed, and its consideration postponed until the next annual session of the Society, which was agreed to.

The resolutions on Vaccination and Vaccine Matter, offered yesterday by Dr. Emerson, were called up by Dr. Worthington, and on his motion agreed to.

The report made yesterday by the Committee on Nomination, was read, on call; and on motion of Dr. Condie it was—

*Resolved,* That the officers therein named be, and they are hereby declared to be the officers of the Society for the ensuing year.

The President elect, Dr. R. La Roche, upon taking his seat, addressed the Society as follows:—

GENTLEMEN OF THE STATE MEDICAL SOCIETY:

In assuming the duties of the office to which you have this day elevated me, I would do injustice to my feelings, were I to fail to express to you my most sincere thanks for the honor thus conferred on me. It is an honor to which I feel I can lay no claim, and which I must owe rather to the partiality of friends than to any merit of mine. Certainly the announcement of your choice was to me most unexpected. Little did I dream that I was ever to be chosen to preside over your deliberations. The duties of the office are among those for which I think myself least qualified, from habit and taste, as well as from want of experience, to perform in a satisfactory manner. I therefore at once appeal to your indulgence; for, without a large share of that virtue, you will, I fear, have many an occasion to regret not having made another selection. But on this subject you have assumed the responsibility. All I can promise is that no effort will be neglected by me, to discharge the duties you have been kind enough to confide to me, to the full extent of my abilities, and with the zeal which the warm interest which I have ever taken in the success of this Society, and in the honor and high standing of our profession, animate me.

On motion of Dr. Condie, the selection of West Chester as the place of meeting in 1857 was approved, and 11 o'clock, A. M., on the last Wednesday in May next, was fixed as the hour at which the next session shall be opened.

On motion of Dr. H. Carpenter, the President was requested to deliver an address at the opening of the next session.

On motion of Dr. Condie, it was—

*Resolved,* That the thanks of the Society be tendered to the retiring President, Dr. Jas. S. Carpenter, for the courteous and impartial manner in which he has presided over the deliberations of the session.

On motion of Dr. Bell, the thanks of the Society were tendered to the other officers of their prompt and efficient discharge of duty.

The following resolutions, proposed by Dr. Wythes, and committed in 1854, and recommended for adoption in 1855, but accidentally overlooked, were, on call of Dr. Kennedy, taken up, and on his motion agreed to:—

*Resolved,* 1. That the several County Medical Societies are recommended to appoint their annual committees, to prepare reports as early as practicable after the meetings of the State Society, and that said committees subdivide themselves respectively into sections, having reference to the different topics of Medical information, namely: pathology and therepeutics, surgery, midwifery, etc.

*Resolved*, 2. Papers read before a county Society should be referred to the appropriate section of the committee, to be used in the preparation of the annual report. Papers of more than ordinary merit or interest, to be reported at length, or an abstract made of them, at the option of the Society.

On motion of Dr. Gries, it was—

*Resolved*, That the thanks of the State Medical Society are hereby presented to the Board of Trustees of the University of Pennsylvania, for their liberality in granting the use of the Hall of the University for its meeting during the present session.

On motion of Dr. H. Carpenter, it was—

*Resolved*, That the thanks of the Society be, and they are hereby tendered to the Philadelphia Press, for their faithful report of each day's proceedings, to the Pennsylvania Institute for the Instruction of the Blind, to the Trustees of the University of Pennsylvania, to the Academy of Natural Science, to Wm. H. Allen, LL. D., President of Girard College for Orphans, to the Philadelphia Hospital for Diseases of the Chest, and others, for the kind invitation extended by them to the Society, to visit their respective institutions.

On motion of Dr. Emanuel, it was—

*Resolved*, That the thanks of this Society be tendered to the Committee of Arrangement, for the handsome manner in which they have been received and entertained during their attendance at this session.

On motion of Dr. Wister, the Society adjourned.

HENRY CARPENTER,
ALFRED L. KENNEDY,
*Recording Secretaries.*

